# HIV-1-neutralizing antibody induced by simian adenovirus- and poxvirus MVA-vectored BG505 native-like envelope trimers

**DOI:** 10.1371/journal.pone.0181886

**Published:** 2017-08-09

**Authors:** Silvia Capucci, Edmund G. Wee, Torben Schiffner, Celia C. LaBranche, Nicola Borthwick, Albert Cupo, Jonathan Dodd, Hansi Dean, Quentin Sattentau, David Montefiori, Per J. Klasse, Rogier W. Sanders, John P. Moore, Tomáš Hanke

**Affiliations:** 1 The Jenner Institute, University of Oxford, Oxford, United Kingdom; 2 Department of Pharmacy and Biotechnology, University of Bologna, Bologna, Italy; 3 The Sir William Dunn School of Pathology, University of Oxford, Oxford, United Kingdom; 4 Department of Surgery, Duke University Medical Center, Durham, North Carolina, United States of America; 5 Department of Microbiology and Immunology, Weill Cornell Medical College, New York, New York, United States of America; 6 The International AIDS Vaccine Initiative, New York, New York, United States of America; 7 Department of Medical Microbiology, University of Amsterdam, Amsterdam, the Netherlands; 8 International Research Center for Medical Sciences, Kumamoto University, Kumamoto, Japan; Shanghai Medical College, Fudan University, CHINA

## Abstract

Rabbits and monkeys immunized with HIV type 1 (HIV-1) native-like BG505 SOSIP.664 (BG505s) glycoprotein trimers are known to induce antibodies that can neutralize the autologous tier-2 virus. Here, we assessed the induction of HIV-1 trimer binding and neutralizing antibody (nAb) titres when BG505s trimers were also delivered by non-replicating simian (chimpanzee) adenovirus and non-replicating poxvirus modified vaccinia virus Ankara (MVA) vaccine vectors. First, we showed that approximately two-thirds and one-third of the trimers secreted from the ChAdOx1.BG505s (C) and MVA.BG505s (M) vaccine-infected cells, respectively, were cleaved and in a native-like conformation. Rabbits were immunized intramuscularly with these vaccine vectors and in some cases boosted with ISCOMATRIX^™^–adjuvanted BG505s protein trimer (P), using CCC, MMM, PPP, CPP, MPP and CMP vaccine regimens. We found that the peak trimer-binding antibody and tier-1A and autologous tier-2 nAb responses induced by the CC, CM, PPP, CPP, MPP and CMP regimens were comparable, although only PPP induced autologous tier-2 nAbs in all the immunized animals. Three animals developed weak heterologous tier-2 nAbs. These results demonstrate that ChAdOx1 and MVA vectors are useful delivery modalities for not only T-cell, but also antibody vaccine development.

## Introduction

Vaccines remain the best solution to ending of the HIV-1/AIDS epidemic. Ideally, a vaccine would induce protective levels of broadly neutralizing antibodies (bnAbs) against multiple HIV-1 variants [1, 2]. At least initially in an immunization regimen, while bnAb induction is sub-optimal, antibody responses may need to be supplemented by effective cytotoxic T-lymphocyte responses [3, 4]. Elicitation of either of these arms of immunity continues to pose major scientific challenges.

The envelope glycoprotein (Env) trimer is the only relevant target for antibodies on HIV-1 virions. bnAbs emerge in 20–30% of HIV-1-infected individuals after two or more years of infection, and multiple lineages have now been isolated [1, 5–19]. In these individuals, bnAbs do not provide clinical benefit because they are slow to mature and once they appear, the viruses can still escape [20–22]. Passive transfer of bnAbs can achieve sterilizing immunity in rhesus macaques, if sufficient concentrations are present at the time of or immediately after the virus challenge [23–30]. For active immunization by Env-based vaccines, the key obstacle is the need to induce bnAbs against tier-2 viruses, which dominate human transmission and are relatively neutralization-insensitive. Nevertheless, increasing understanding of the Env structure and its glycan shield, and the ability to follow virus and antibody co-evolution during infection and evolutionary convergence of bnAb specificities among some patients [31, 32] are cumulative, if gradual, steps towards the induction of bnAbs.

One approach to the problem of inducing bnAbs is the design platform represented by recombinant native-like soluble trimers, for which the prototype is the BG505 SOSIP.664 construct [33]. These trimers, from hereon referred to as BG505s, consist of furin-cleaved gp120 and gp41 ectodomains, are stabilized by an inter-subunit disulfide bond and a point substitution in gp41, and display multiple human bnAb epitopes [33–36]. In rabbit and macaque immunizations, BG505s trimers adjuvanted with ISCOMATRIX^™^ induced cross-reactive nAbs against tier-1 viruses and nAbs against the autologous BG505.T332N tier-2 virus, although nAbs against heterologous tier-2 viruses were only elicited sporadically and at low titres [37–39]. The BG505s trimer design is capable of improvement from the perspectives of trimer engineering and delivery [40].

Strategies combining simian (chimpanzee) adenoviruses and poxvirus modified vaccinia virus Ankara (MVA) into heterologous prime-boost regimens have induced both high-titre antibody [41–46] and T-cell [47–50] responses against the shared transgene products. Here, we assessed the *in vivo* expression of the BG505s trimers from the viral vector-infected cells, and immunogenicity of the viral vaccines alone and with boosting by adjuvanted BG505s trimers. We measured the titres of trimer-binding and virus-neutralizing Abs in the various regimens. We report that vector-expressed BG505s trimers are able to induce nAb titres that can be increased by boosting with soluble protein trimers.

## Materials and methods

### Construction of ChAdOx1.BG505s

The E1/E2-deleted vaccine vector ChAdOx1 is derived from chimpanzee adenovirus isolate Y25 of group E [51]. For the generation of recombinant ChAdOx1, the BG505 SOSIP.664 gene [33] was inserted into the E1 locus of the adenovirus genome (integrated in bacterial artificial chromosome) under the control of human cytomegalovirus immediate early promoter. The ChAdOx1.BG505s vaccine was rescued by transfection of HEK 293A T-Rex cells with the PmeI-excised ChAdOx1.BG505s genome. The transgene presence and absence of contaminating empty parental adenovirus in the virus stock was confirmed by PCR, the vaccine was titred and stored at –80°C until use.

### Construction of MVA.BG505s

To generate MVA.BG505s, the BG505 SOSIP.664 gene [33] was subcloned under control of the modified H5 promoter. Through homologous recombination in chicken embryo fibroblast cells, the expression cassette was directed into the thymidine kinase locus of the MVA genome. Initially co-inserted green fluorescent protein marker was removed by trans-dominant recombination to generate markerless vaccine MVA.BG505s [52]. The virus was plaque-purified, expanded, purified on a 36% sucrose cushion, titred and stored at –80°C until use.

### Production of BG505s trimers

BG505 SOSIP.664 trimers were produced and purified as reported previously [33, 36, 53]. Proteins were expressed in HEK 293T cells (i.e. have a human cell glycosylation profile) by transient transfection and purified using a 2G12 mAb affinity column followed by size-exclusion chromatography and stored at -80°C until use.

### *In vitro* expression of BG505s trimers from vaccine-infected cells

HEK 293T cells were grown in 6-well plates to 70% confluence and infected with either ChAdOx1.BG505s or MVA.BG505s at a multiplicity of infection (MOI) of 5 or 50 for 1 h. The cells were then washed and grown for 24 or 48 h, and the culture supernatants were harvested and stored in 10% FBS at 4°C until use. To monitor the release of trimers into the cell culture supernatants, we developed a two-stage capture ELISA protocol.

In the first stage, high-binding ELISA plates (Greiner Bio-One) were coated with 4 μg/ml of capture mAb 2G12 in PBS at 4°C overnight. After washing 3x with PBS+0.05% Tween 20 (PBST), the wells were blocked using 200 μl/well of 2% BSA (Sigma) in PBST at room temperature (RT) for 1 h and washed 1x with PBST. A titration curve was prepared by diluting purified BG505s trimers of a known concentration in culture supernatant from untransfected cells. The Env proteins present in the various culture supernatants were captured onto the ELISA plates via the 2G12 mAb at RT for 2 h and the plates were washed 3x in PBST. Bound trimer was detected with biotinylated mAb VRC01, washed 3x with PBST, and labelled with horseradish peroxidase (HRP)-conjugated streptavidin (1:2000; GE Healthcare). Following 3 PBST washes, the colorimetric endpoint was obtained using the 1-Step ultra tetramethylbenzidine substrate (Thermo Scientific) until a signal of ~1.5 OD_450_ units was generated. Color development was stopped with 0.5 M sulfuric acid, and the 450-nm and 570-nm absorptions were recorded. After background subtraction, a “one-site-specific-binding-with-hill-slope” curve was fitted to the control titration curve using Prism v6 (GraphPad Software) and the concentrations of Env proteins present in the various culture supernatants were extrapolated.

In the second stage, the capture ELISA involved the same protocol, but with either 2G12 or PGT151 as the capture mAb and biotinylated PGT145 as the detection mAb. A series of samples with the same overall Env concentration were prepared, which were made up of purified trimeric and monomeric BG505 species mixed at different ratios from monomer only (0% correctly folded) to trimer only (100% correctly folded). Data analysis was performed as in the first stage.

### Immunization of rabbits by modified HIV-1 Env

Rabbit immunization and blood sampling was carried out at MediTox, s.r.o. (The Czech Republic). The rabbits (10- to 14-week-old randomized male/female HYLA; http://www.eurolap.fr) were given either 5x10^10^ virus particles of ChAdOx1.BG505s, 10^8^ plaque-forming units of MVA.BG505s or 30 μg of BG505s trimer protein adjuvanted in 75 units of ISCOMATRIX^™^, a saponin-based adjuvant obtained from CSL (Kankakee, Illinois, USA), per dose. All doses were administered intramuscularly. Serum was isolated from blood collected biweekly between weeks 0 and 38, and stored at -80°C until use. All animals were observed for clinical signs, morbidity or mortality once a day during the acclimatization, immunization and wash-out periods. Nine animals died during the study (S1 Table) for vaccine-unrelated causes as judged by the study vet. The study was carried out in full compliance with EMA, The European Agency for the Evaluation of Medicinal Products, Human Medicines Evaluation Unit, CPMP/SWP/465/95 and relevant Standard Operating Procedures of MediTox s.r.o.

### Capture ELISA for quantifying binding Abs in rabbit sera

The 2G12 mAb (2.0 μg/ml) was coated onto ELISA plates (Greiner Bio-One) at 4°C during an overnight incubation. The wells were washed 3x in PBS-T and blocked with 2% BSA in PBS plus 0.05% Tween. After washing 1x in PBS-T, BG505s (0.2 μg/ml) was added to all wells at RT for 2 h. After washing 3x in PBS-T, serial 5-fold dilutions of sera starting from 1:100 were added to wells at RT for 2 h, the plates were washed 5x in PBS-T, incubated with 50 μl/well of secondary mouse anti-rabbit-HRP antibody (Millipore) diluted 1:15000 in 1% BSA in PBS at RT for 1 h, and washed 3x with PBS-T. The colorimetric reaction was carried out as described above. Note that this ELISA format was not identical to the one used in previous rabbit immunization studies [33–36]. Hence, the absolute titres may not be comparable across experiments.

### HIV-1-neutralization assay

A validated TZM-bl neutralization assay using Env-pseudotyped viruses was described previously [54]. The test panel used here included the tier-1 viruses MN.3 and MW965.26, the autologous tier-2 virus BG505ΔCT/T332N and a global panel of heterologous tier-2 viruses (25710–2.43, TRO.11, BJOX0020_00.03.2, X1632-S2-B10, Ce1176_A3, 246-F3_C10_2, CH119.10, Ce70301_0217_B6 and CNE55) [55]. The glycan knock-in mutant viruses BG505ΔCT/T332N.S241N, BG505ΔCT/T332N.P291T and BG505ΔCT/T332N.S241N.P291T were used to assess the importance of glycan holes for neutralization [38]. For additional information on the assay and related protocols, see www.hiv.lanl.gov/content/nab-reference-strains/html/home.htm. No signal was detected when a murine leukemia virus-pseudotyped virus was used to estimate non-specific neutralization.

### Statistical analysis

Statistical analyses were performed using Prism v6 (GraphPad Software). Two-group comparisons were performed with Mann-Whitney U test. Multi-group comparisons were performed with Kruskal-Wallis test and Dunn’s multiple comparison post-test. Correlation between neutralization of different viruses was analysed by using the Spearman nonparametric method. A *P* value (two-tailed) <0.05 was considered significant.

## Results

### Construction of the ChAdOx1.BG505s and MVA.BG505s vaccines

Two non-replicating virus-vectored vaccines ChAdOx1.BG505a and MVA.BG505s were constructed as described previously [52, 56]. The non-replicating ChAdOx1 vector is derived from a group E adenovirus Y25 with low human seroprevalence [51]. The parental non-replicating MVA originates directly from Professor Mayr, passage 575 dated 14-12-1983. Both vaccines express BG505 SOSIP.664 gp140 (ref. [33]) with the natural HIV-1 leader sequence replaced by that of the human tissue plasminogen activator; they do not include a gene expressing the furin protease, but instead rely on endogenous furin-family proteases for trimer maturation [57].

### *In vitro* secreted BG505s trimers from vaccine-infected human cells maintain their integrity

**Fig 1:**
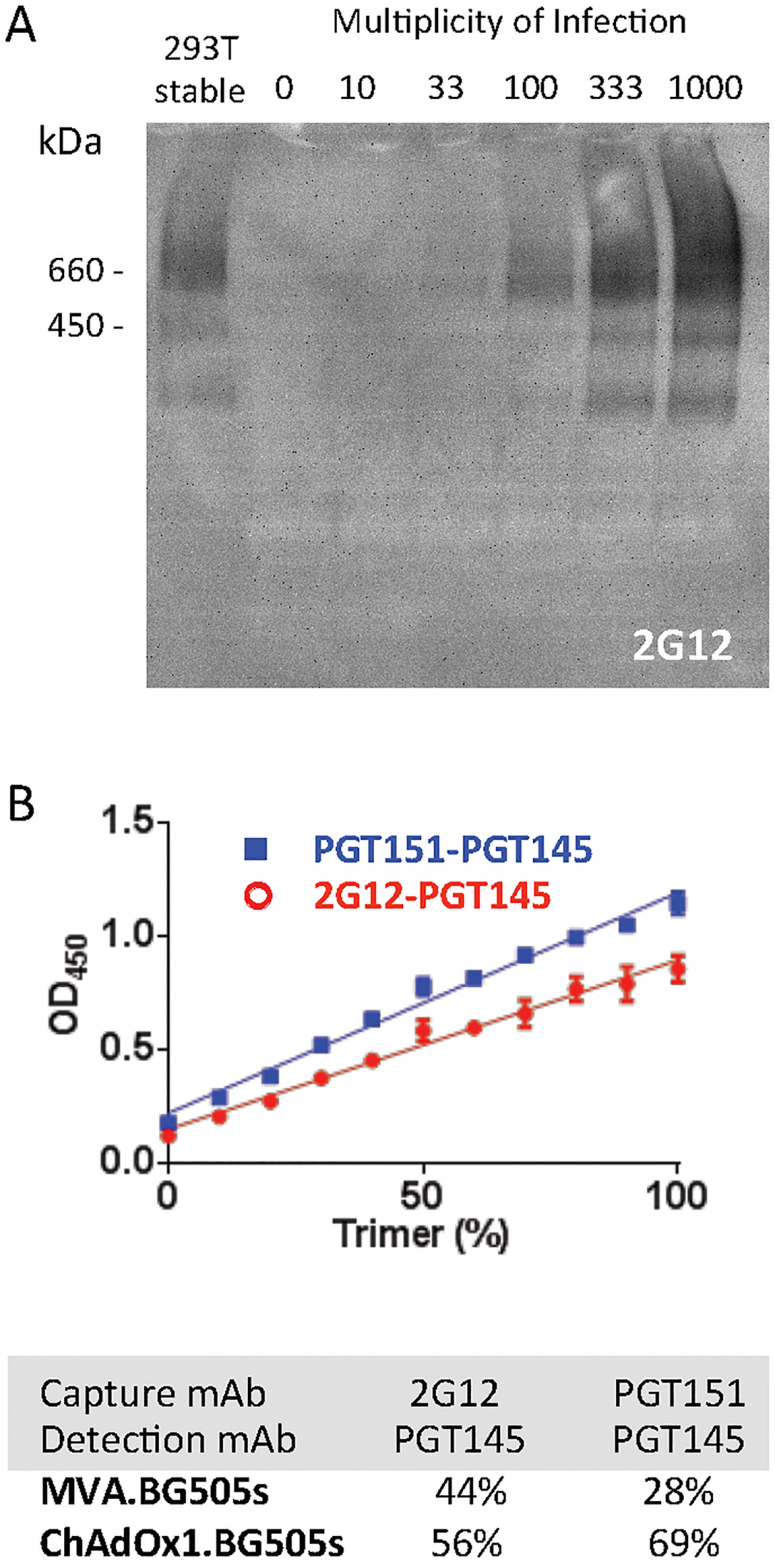
Expression of BG505s trimers in tissue culture. (A) Monolayers of HEK 293T cells were infected with ChAdOx1.BG505s at the indicated MOIs for 48 h. The proteins in the tissue culture supernatants were separated by non-denaturing polyacrylamide gel electrophoresis and transferred onto a nitrocellulose membrane. BG505 Env proteins were detected using the glycan-specific 2G12 mAb as the primary antibody. As a positive control, supernatant from a HEK 293T stable cell line that expresses fully cleaved BG505s trimers was analysed in the first lane. The relative molecular masses of 600 and 450 kDa are indicated. (B) To assess the percentage of correctly folded trimers in the ChAdOx1.BG505s- and MVA.BG505s-infected cell culture supernatants, the ELISA plates were first coated with either 2G12 or PGT151 Env-specific bnAbs and the captured native-like, furin-cleaved trimers were detected using PGT145 as the secondary mAb. To generate the standard curve used for trimer quantification (top), gp140 monomers and trimers purified from stably transfected cells were mixed at different ratios, but at a constant total concentration. The latter values were determined using a different, non-trimer specific capture ELISA (see Methods section). The percentages of native-like BG505s trimer present in the virus-infected cell culture supernatants are shown for the indicated antibody combinations (bottom).

To assess whether native-like BG505s trimers could be expressed from the virus vectors, we used cultured human HEK 293T cells as a model system. The cells were infected with each virus at multiplicity of infection (MOI) of 0 (no infection), 10, 33, 100, 333 and 1000 for 48 h. We first analysed the BG505 Env proteins released into the culture supernatants by non-denaturing gel electrophoresis and Western blotting using the glycan-specific 2G12 mAb as the detection antibody (Fig 1A). A 2G12-reactive protein band of relative molecular mass of ~600 kDa that corresponds to an Env trimer was detectable in the supernatants of cells infected with the ChAdOx1.BG505s virus at MOIs >100 (Fig 1A), but we were unable to detect any Env protein expression by Western blot at any MOI and at any time point in the MVA.BG505s-infected cell supernatants. This is probably because the poxvirus promoter is much weaker than the cytomegalovirus immediate early promoter employed in the recombinant adenovirus.

Next, we assessed whether appropriately folded trimers were present in the culture supernatants by using a capture ELISA based on the properties of the PGT145 and PGT151 bnAbs. PGT145 recognizes an apex-specific quaternary epitope and PGT151 a gp120-gp41-specific quaternary epitope, and both bind only to native-like, furin-cleaved trimers. In this experiment, HEK 293T cells were infected with either vaccine at MOIs of 5 and 50 for 24 and 48 h. Using a 2G12-capture, biotinylated-VRC01 detection ELISA format, which does not distinguish between native-like trimers and other forms of Env, we estimated that the highest overall level of Env expression measured in the ChAdOx1.BG505s cultures was ~360 ng/ml at 48 h after infection at an MOI of 50, whereas for the MVA.BG505s cultures, the maximum expression at the same 48-h time-point was only ~4.5 ng/ml at an MOI of 5. The ~100-fold greater expression in the ChAdOx1.BG505s culture compared to MVA.BG505s concurs with the Western blotting data described above. Next, we used ELISA to estimate the fraction of native-like trimers in the culture supernatants by combining either 2G12- or PGT151-capture with biotin-PGT145 detection, thereby making use of the quaternary epitope specificity of these bnAbs. In a control experiment, we mixed purified “non-native” monomeric BG505 SOSIP.664 proteins with purified native-like trimers at different ratios, but a constant overall Env concentration. The use of the mixtures showed that there was a linear relationship between the proportion of native-like trimers in the sample and the ELISA signal (OD_450_). We used the resulting titration curve to estimate that approximately two-thirds and one-third of the total Env proteins in the ChAdOx1.BG505s and MVA.BG505s supernatants, respectively, were appropriately folded trimers (Fig 1B).

### ChAdOx1.BG505s delivers a stronger prime than an adjuvanted protein trimer

**Fig 2:**
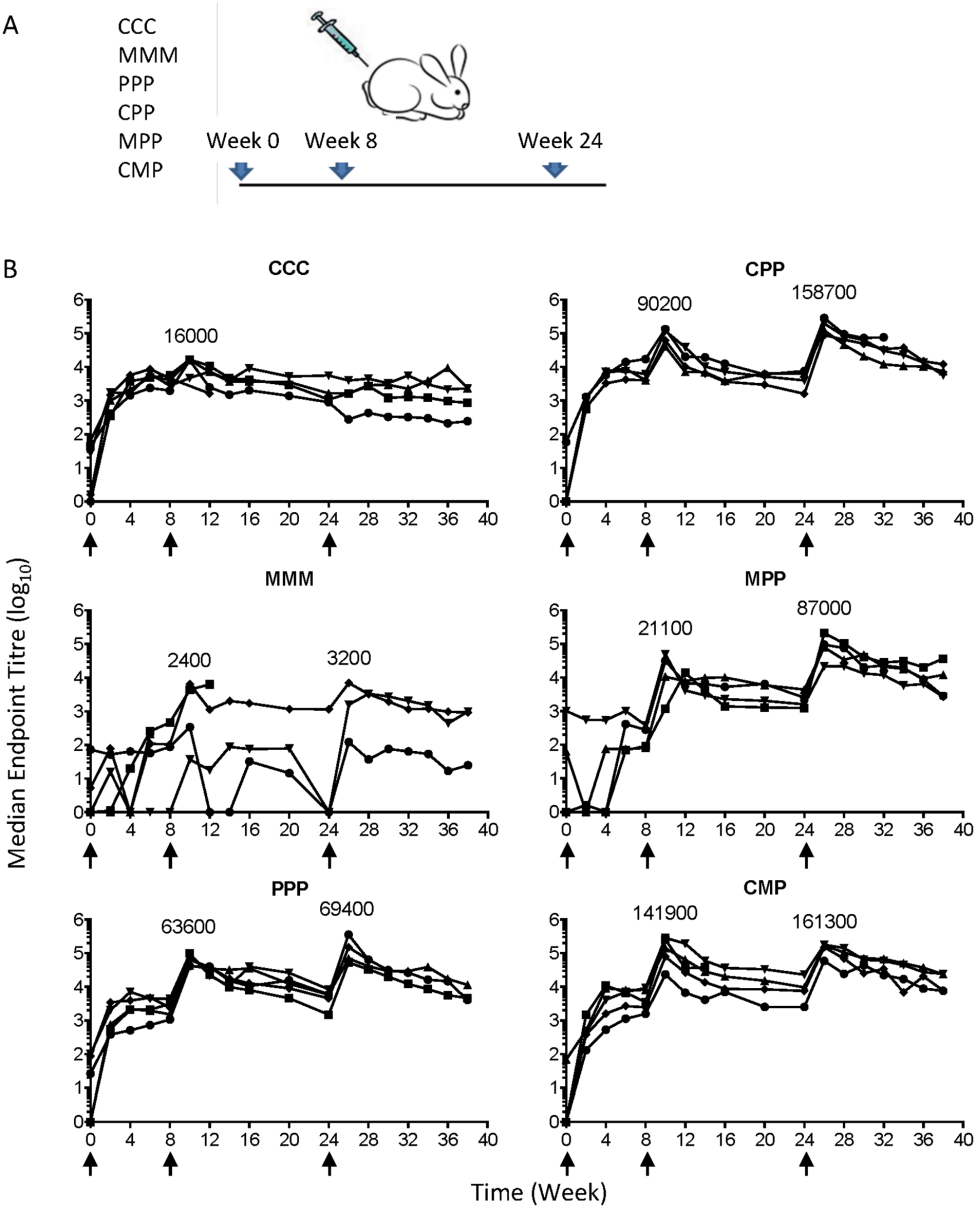
Kinetics of BG505s trimer-binding antibody responses induced in rabbits by various immunization regimens. (A) Groups of 5 rabbits were immunized with ChAdOx1.BG505s (C), MVA.BG505s (P) and ISCOMATRIX^™^-adjuvanted BG505s protein trimer (P) candidate vaccines, as depicted. The animals were immunized at weeks 0, 8 and 24 (arrows), and bled regularly. (B) Reciprocal endpoint titres of the BG505s trimer-binding antibodies in rabbit sera were determined by capture ELISA. The trimer-binding antibody titres at each time point are shown for individual rabbits, and the median values for group-peak time points are shown above each peak. Note that the time points at which the peak titres were measured differed among the rabbits in each group, and that some rabbits died for protocol-unrelated reasons (S1 Table).

To assess immunogenicity, six groups of rabbits were immunized with the ChAdOx1.BG505s (C), MVA.BG505s (M) or HEK 293T-produced, ISCOMATRIX^™^-adjuvanted BG505s protein trimer (P) vaccines in either homologous or heterologous regimens of CCC, MMM, PPP, CPP, MPP and PPP (Fig 2A). For the PPP group, the 0-8-24 week immunization protocol, the 30-μg amount of trimer per dose, its source, and the adjuvant were all similar to those used in previous rabbit studies [37–39]. Serum samples were drawn at 2- to 4-week intervals until week 38, which was 14 weeks after the last immunization. In the first instance, we used a capture ELISA to monitor the induction kinetics of antibodies reactive with the BG505s trimer (Fig 2B, S1 Table). Comparing the peak trimer-binding titres to gauge the antibody response to a single C, M or P dose, the vaccine modalities ranked in the order of C > P > M with median reciprocal titres of 6300 (n = 13), 3100 (n = 5) and 100 (n = 9), respectively (Table 1 and S1 Table). The differences in peak responses induced by a single C, M and P administration were highly significant (*P* < 0.0001) (Kruskal-Wallis test), as was C vs P (*P* < 0.0001) (Dunn’s multiple comparison test).

**Table 1:** Peak reciprocal anti-trimer Ab end-point titres in rabbit sera, ranked by immunization group.

Rank	Regimen	Median	Range	n
1	CMP	161300	58900–179400	4
2	CPP	158700	88400–285600	4
3	CM	141900	23700–287500	5
4	CP	90200	40300–133600	4
5	MPP	87000	21600–210200	4
6	PPP	69400	52300–287500	5
7	PP	63600	41600–98500	5
8	MP	22800	10500–49600	4
9	CC	16000	9200–16700	4
10	C	6300	1600–17300	13
11	CCC	4200	400–9400	4
12	MM	3400	100–6400	4
13	MMM	3300	100–7000	3
14	P	3100	1100–7000	5
15	M	100	0–1000	9

### PPP is the most immunogenic homologous vaccination regimen

Examining first the single-modality regimens, the binding titre for repeated ChAdOx1.BG505s immunizations peaked after the second dose (CC) at a median reciprocal titre of 16000; the third dose was inefficient, likely due to the anti-vector immunity. For the MVA.BG505s-only regimen, the initial titres were very low, but continued to increase with boosting; thus, the third vaccine administration (MMM) boosted the titres to a median of 3300. The sequential delivery of adjuvanted protein trimer (PPP) kept boosting the Ab responses and peaked at 69400 (Table 1).

### Heterologous-modality and protein-only regimens induce similar BG505s-binding antibody titres

The best regimen after two immunizations was CM, with a median endpoint reciprocal titre of 141900, ~2-fold higher than the value of 63600 for the PP regimen (Table 1). Based on the peak titres recorded after the third immunization, the rank order for the various regimens was CMP > CPP > MPP > PPP with respective median reciprocal titres of 161300, 158700, 87000 and 69400 (non-significant using Kruskal-Wallis test) (Fig 2B, Table 1). Thus, from the perspective of inducing ELISA-reactive anti-trimer antibodies, priming with one or more virus vector (i.e., CMP or CPP) increases the subsequent response to an adjuvanted-protein boost by over 2-fold compared to the protein-only regimen (PPP).

### Broad tier-1A and narrow-specificity tier-2 nAb responses

To assess HIV-1 neutralization, we used a validated TZM-bl cell assay [54] with the tier-1A viruses MN.3 and MW965.26, the BG505ΔCT/T332N vaccine strain and a Global Virus Panel of tier-2 viruses (Table 2). For the MMM, PPP, CPP, MPP and CMP immunization groups, we measured nAb responses using sera from week-26, i.e. 2 weeks after the third/final immunization (Fig 2A). We also included sera from the CCC and CMP groups that were obtained at week 10, which is 2 weeks after the second vector immunization. At that time, these animals had not received a BG505s trimer (P) boost, so for clarity these week-10 sera are designated as CC and CM, respectively. The eight analytical groups included in the nAb comparison sub-study and the resulting titre values are outlined in Table 2. BG505s trimers are known to induce a wide range of autologous nAb titres (from 50 to >10000) in individual rabbits, and tier-2 nAb titres also vary considerably [36, 39]. Such variation limits the power of any comparison between immunization groups that involve 4–5 rabbits. We have therefore generally refrained from making statistical comparisons between the various analytical groups.

**Table 2:** Neutralization of tier-1 and tier-2 viruses in TZM-bl cell assay^a^.

				Tier 1A		Tier 2									
				Clade B	Clade C	Clade A	Clade C	Clade B	CRF07 BC	Clade B	Clade C	Clade AC	CRF07 BC	Clade A	CRF01 AB
Regimen	Animal ID	Bleed Week	Negative Control	MN.3	MW965.26	BG505ΔCT/T332N	25710–2.43	TRO.11	BJOX002000.03.2	X1632-S2-B10	Ce1176 A3	246-F3 C10 2	CH119.10	Ce703010217 B6	CNE55
**CC**	M17	10	<20	**558**	**2819**	**73**	<20	<20	<20	<20	<20	<20	<20	<20	<20
	M19	10	<20	**554**	**895**	**70**	<20	<20	<20	<20	<20	<20	<20	<20	<20
	M23	10	34	**584**	**1207**	40	32	32	28	35	28	35	40	27	24
	F2	10	<20	**714**	**802**	**33**	<20	<20	<20	<20	<20	<20	<20	<20	<20
	F21	10	<20	**635**	**1690**	<20	<20	<20	<20	<20	<20	<20	<20	<20	<20
median			<20	**584**	**1207**	**40**	<20	<20	<20	<20	<20	<20	<20	<20	<20
**CCC**	M17	26	<20	<20	**189**	<20	<20	<20	<20	<20	<20	<20	<20	<20	<20
	M19	26	<20	<20	**76**	<20	<20	<20	<20	<20	<20	<20	<20	<20	<20
	M23	26	<20	<20	<20	**36**	<20	<20	<20	<20	<20	<20	<20	<20	<20
	F2	26	<20	<20	**65**	<20	<20	<20	<20	<20	<20	<20	<20	<20	<20
median			<20	<20	**71**	<20	<20	<20	<20	<20	<20	<20	<20	<20	<20
**MMM**	M8	26	<20	<20	<20	<20	<20	<20	<20	<20	<20	<20	<20	<20	<20
	F20	26	<20	<20	**710**	<20	<20	<20	<20	<20	<20	<20	<20	<20	<20
	F31	26	<20	<20	<20	**311**	<20	<20	<20	<20	<20	<20	<20	<20	<20
median			<20	<20	<20	<20	<20	<20	<20	<20	<20	<20	<20	<20	<20
**PPP**	M10	26	<20	**496**	**5296**	**559**	<20	<20	<20	<20	<20	<20	<20	<20	<20
	M12	26	<20	**112**	**2171**	**925**	<20	<20	<20	<20	<20	<20	<20	<20	<20
	M27	26	<20	**61**	**2385**	**45**	<20	<20	<20	<20	<20	<20	<20	<20	<20
	F4	26	<20	**121**	**13856**	**150**	**80**	**29**	<20	<20	**77**	<20	<20	<20	<20
	F13	26	<20	**1714**	**33189**	**41**	<20	<20	<20	<20	<20	<20	<20	<20	<20
median			<20	**117**	**5296**	**150**	<20	<20	<20	<20	<20	<20	<20	<20	<20
**CPP**	M7	26	<20	**235**	**8618**	<20	<20	<20	<20	<20	<20	<20	<20	<20	<20
	F5	26	<20	**879**	**1397**	**151**	**27**	**21**	<20	<20	**39**	<20	<20	<20	<20
	F6	26	<20	**325**	**13309**	**553**	<20	<20	<20	<20	<20	<20	<20	<20	<20
	F22	26	<20	**87**	**8532**	<20	<20	<20	<20	<20	<20	<20	<20	<20	<20
median			<20	**280**	**8575**	**76**	<20	<20	<20	<20	<20	<20	<20	<20	<20
**MPP**	M9	26	<20	**467**	**1770**	**125**	<20	<20	<20	<20	<20	<20	<20	<20	<20
	M15	26	<20	**521**	**8599**	<20	<20	<20	<20	<20	<20	<20	<20	<20	<20
	M18	26	<20	**1026**	**6008**	**400**	**42**	<20	<20	<20	**45**	<20	<20	<20	<20
	F29	26	<20	**137**	**847**	**847**	<20	<20	<20	<20	<20	<20	<20	<20	<20
median			<20	**494**	**3889**	**263**	<20	<20	<20	<20	<20	<20	<20	<20	<20
**CM**	M11	10	<20	**1130**	**940**	<20	<20	<20	<20	<20	<20	<20	<20	<20	<20
	F1	10	<20	**20545**	**11454**	<20	<20	<20	<20	<20	<20	<20	<20	<20	<20
	F3	10	<20	**540**	**2770**	**90**	<20	<20	<20	<20	<20	<20	<20	<20	<20
	F26	10	<20	**6558**	**2732**	**28**	<20	<20	<20	<20	<20	<20	<20	<20	<20
	M16	10	<20	**2070**	**2071**	**245**	<20	<20	<20	<20	<20	<20	<20	<20	<20
median			<20	**2070**	**2732**	**28**	<20	<20	<20	<20	<20	<20	<20	<20	<20
**CMP**	M11	26	<20	**468**	**1470**	<20	<20	<20	<20	<20	<20	<20	<20	<20	<20
	F1	26	<20	**343**	**8624**	**37**	<20	<20	<20	<20	<20	<20	<20	<20	<20
	F3	26	<20	**397**	**6355**	**2096**	<20	<20	<20	<20	<20	<20	<20	<20	<20
	F26	26	<20	**6199**	**24627**	**1011**	<20	<20	<20	<20	<20	<20	<20	<20	<20
median			<20	**432**	**7490**	**524**	<20	<20	<20	<20	<20	<20	<20	<20	<20

^a^—Values are the serum dilution at which relative luminescence units (RLUs) were reduced 50% compared to virus control wells (no test sample). Highlighted bold values are considered positive for neutralization based on the criterion of signal ≤3x the signal of that sample against the MLV-pseudotyped negative-control virus.

The tier-1 nAb titres against the MN.3 and MW965.26 viruses were very low for the CCC and MMM regimens at week 26 (median titres <100). These minimal responses were markedly lower than those seen in the CC and CM groups at week 10 (584/1207 and 2070/2732, respectively, for the MN.3/MW965.26 viruses). The observations imply that the vector-only regimens induce early tier-1 nAb responses that are either not sustained or are not reboosted by a third immunization at week 24, perhaps due to anti-vector immunity. For the PPP, CPP, MPP, CMP regimens that involved one or more BG505s trimer immunizations the tier-1A nAb titres at week-26 were broadly comparable, the medians for the two test viruses differing by only ~2-fold. Thus, the CC, CM, CPP, MPP, CMP and PPP regimens induced similar levels of nAbs against the two tier-1 viruses.

The CC, CM (week 10) and CCC (week 26) regimens induced only sporadic and low (<75) autologous tier-2 nAb titres. Most of the rabbits receiving the PPP, CPP, MPP and CMP regimens developed autologous tier-2 nAb responses at week 26. Within the limitations imposed by the small group sizes, it seems reasonable to conclude that the CMP, MPP and PPP regimens were comparably effective at inducing nAbs against the autologous tier-2 virus.

Finally, sera from three rabbits, one from the PPP regimen (animal F4), one from CPP (animal F5) and one from MPP (animal M18), weakly neutralized (titres <100) 2 or 3 viruses in the tier-2 Global Panel. The viruses neutralized were TRO.11 (clade B), 25710–2.43 (clade C) and Ce1176_A3 (clade C). It is not possible to identify any pattern in these occasional low-level responses, although we note that all the rabbits had received a trimer boost.

### Autologous tier-2 nAb responses are to epitopes involving a glycan hole at residues 241 and 289

We have reported that a PPP regimen induces autologous nAbs in rabbits that often, but not invariably, target a hole in the glycan shield created by the lack of a glycan at position 241 and/or 289 [38]. Thus, BG505.T332N virus mutants with glycans knocked in at these positions via S241N and P291T substitutions could have reduced or absent sensitivity to neutralization by rabbit sera [38]. Therefore, we tested 24 sera from the present study against the same BG505ΔCT/T332N.S241N, BG505ΔCT/T332N.P291T and BG505ΔCT/T332N.S241N.P291T glycan hole knock-in viruses used previously. No serum that was unable to neutralize the parental BG505.T332N virus could neutralize any of the mutant viruses, and both the single and double glycan-knock-in mutations resulted in markedly reduced autologous neutralization titres (Table 3). Thus, the nAb specificities induced in the present study are generally consistent with those we have described previously for the PPP regimen [38].

**Table 3:** Antibody recognition of glycan holes^a^.

Regimen	Animal ID	Bleed Week	BG505ΔCT T332N	BG505ΔCT T332N.S241N	BG505ΔCT T332N.P291T	BG505ΔCT T332N.S241N.P291T
	M17	26	nd	nd	nd	nd
**CCC**	M19	26	<20	nd	nd	nd
	M23	26	<20	<20	<20	<20
	F2	26	<20	<20	<20	<20
	M8	26	<20	<20	<20	<20
**MMM**	F20	26	<20	<20	<20	<20
	F31	26	**155**	**68**	**45**	**53**
	M10	26	**247**	<20	<20	<20
**PPP**	M12	26	**743**	**760**	**614**	**484**
	M27	26	<20	<20	<20	<20
	F4^b^	26	**39**	<20	**23**	<20
	F13	26	<20	<20	<20	<20
	M7	26	<20	nd	nd	nd
**CPP**	F5^b^	26	**74**	**51**	**44**	**58**
	F6	26	**304**	**261**	**184**	**128**
	F22	26	<20	<20	<20	<20
	M9	26	**102**	**68**	**62**	**54**
**MPP**	M15	26	<20	<20	<20	<20
	M18^b^	26	**329**	<20	<20	<20
	F29	26	**405**	**30**	**29**	**26**
	M11	26	<20	<20	<20	<20
**CMP**	F1	26	<20	<20	<20	<20
	F3	26	**2590**	**2381**	**1442**	**1237**
	F26	26	**304**	<20	<20	<20

^a^—Values are the serum dilution at which relative luminescence units (RLUs) were reduced 50% compared to virus control wells (no test sample). Highlighted bold values are considered positive for neutralization based on the criterium of signal ≤3x the signal of that sample against the MLV-pseudotyped negative-control virus;

^b^—animals neutralized 2 to 3 tier-2 viruses (Table 2); nd—not done due to lack of sample.

## Discussion

Developing a vaccine that induces Abs broadly neutralizing globally transmitted HIV-1 variants is a major goal of HIV/AIDS research, but remains a substantial scientific challenge. In the present work, we constructed novel vaccines vectored by chimpanzee adenovirus ChAdOx1 (C) and MVA (M) that express BG505 SOSIP.664 (BG505s) Env proteins and assessed systematically their immunogenicity in homologous and heterologous vaccine regimens that included ISCOMATRIX^™^-adjuvanted trimer proteins (P). The protein-only (PPP) regimen served as the comparator arm, as this immunization method has been tested previously [37–39]. Overall, viral vectors combined sequentially with adjuvanted BG505s trimers induced similar antibody responses to the PPP regimen.

Gp160 expressed within HIV-1-infected cells is almost always fully cleaved into the gp120 and gp41 subunits by the action of a furin-family protease [36]. Also SOSIP.664 trimers must be cleaved to adopt a stable and native-like structure. For *in vitro* SOSIP.664 trimer production by transient transfection or in stable CHO or HEK 293T cell lines, furin co-transfection is used to increase the extent of the recombinant gp140 cleavage. This compensates for the abnormally high Env expression levels driven by strong promoters relative to natural HIV-1 infection and a quite poor expression of furin-family proteases by the cells commonly used for trimer production. The extent to which gp140 is cleaved when expressed from virus vectors *in vivo* is unknown and the answer is not readily obtainable. Comparative immunogenicity experiments using various Env protein immunogens that *in vitro* do or do not require furin co-expression for the trimers native-like conformation, such as NFL, gp140-sc and UFO, would be required [40]. Here, we analyzed the Env proteins secreted from vaccine-infected HEK 293T cells. Even though neither of the ChAdOx1.BG505s or MVA.BG505s vaccines co-delivered a furin gene, two-thirds and one-third of the Env proteins in the supernatants, respectively, were native-like trimers as judged by their expression of quaternary (trimer-specific) epitopes. Furthermore, the immunogenicity data from our experiments clearly show that a substantial proportion of the expressed SOSIP.664 trimers must have been appropriately cleaved, and hence native-like, when expressed in the rabbits, because otherwise we would not have expected to induce an autologous nAb response. Thus, viral vectors can drive the production of native-like BG505 SOSIP.664 trimers *in vitro*, although other, non-native forms of Env are also present, and the resulting trimers are immunogenic in rabbits.

We and others have shown that viral vectors including simian adenoviruses and MVA can induce robust CD4^+^ and CD8^+^ T-cell responses particularly in heterologous prime-boost regimens [47–50]. Here, the rabbit data suggest equal trimer immunogenicity of the virus vector and protein-only regimens. This might be contributed by the likely strong induction of an Env-specific CD4^+^ T-cell help. For future regimens aiming to induce both effective CD8^+^ T cells and bnAbs in parallel and without mutual interference to achieve maximum benefit to the vaccine recipients [1, 3, 4], vaccination regimens may need to be rationally developed to optimize e.g. spatial or temporal separation of the T- and B-cell vaccine administration [58].

It seems unlikely that any single immunogen/Env shape can elicit bnAbs, because their broad specificity evolves from strain-specific Abs through multiple cycles of virus escape and Ab affinity maturation [16, 59, 60]. To mimic this process in the vaccine context may require more complex strategies such as the use of immunogens that engage the germline bnAb precursors [61, 62] followed by guided Ab affinity maturation by sequential isolates from infected individuals who developed bnAbs [31, 63–65]. As virus-vectored vaccines are cheaper to manufacture than protein trimers, they may play a role in delivering candidate Env immunogens in such strategies.

## Supporting information

S1 Table Reciprocal end-point titres of BG505s trimer-binding sera induced in individual rabbits. (PDF)
