# Correction: HIV-1-neutralizing antibody induced by simian adenovirus- and poxvirus MVA-vectored BG505 native-like envelope trimers

**DOI:** 10.1371/journal.pone.0293148

**Published:** 2023-10-12

**Authors:** Silvia Capucci, Edmund G. Wee, Torben Schiffner, Celia C. LaBranche, Nicola Borthwick, Albert Cupo, Jonathan Dodd, Hansi Dean, Quentin Sattentau, David Montefiori, Per J. Klasse, Rogier W. Sanders, John P. Moore, Tomáš Hanke

There are errors in the Funding section. The correct Funding statement is: The work is jointly funded by the Medical Research Council (MRC) UK and the UK Department for International Development (DFID) under the MRC/DFID Concordat agreements (G1001757 and MR/N023668/1); National Institutes of Health grant P01 AI100657; National Institutes of Health grant UM1-AI100645 to the Duke Center for HIV/AIDS Vaccine Immunology-Immunogen Discovery (CHAVI-ID)—subcontract 210782; National Institutes of Health contract HHSN27201100016C; the European Union’s Horizon 2020 research and innovation programme under grant agreement No 681137; T.H. and Q.S. are the Jenner Institute Investigators, and Q.S. is a James Martin Senior fellow. The funders had no role in study design, data collection and analysis, decision to publish, or preparation of the manuscript.
