# Supplementary material for: HIV-1-neutralizing antibody induced by simian adenovirus- and poxvirus MVA-vectored BG505 native-like envelope trimers
Source: PLoS One. 2017 Aug 9;12(8):e0181886. doi: 10.1371/journal.pone.0181886 (PMC5549892; doi:10.1371/journal.pone.0181886)
Supplement: S1 Table — (PDF) [file pone.0181886.s001.pdf]

S1 Table. Reciprocal end-point titres of BG505s trimer-binding sera induced in individual rabbits.

		G1 CCC					
	Rabbit	M17	M19	M23	F2	F21	median
Vacc 1	Week 0	0	0	100	0	2	0
	Week 2	400	300	1000	1800	1500	1000
	Week 4	1500	3800	2100	1600	5700	2100
	Week 6	2400	4800	5100	6300	8600	5100
Vacc 2	Week 8	2000	5800	4200	2800	4600	4200
	Week 10	15800	16700	15300	4800	Clotted	15550
	Week 12	2500	10700	7100	7300	1600	7100
	Week 14	1500	4700	3700	4000	Died	3850
	Week 16	2000	4200	3700	9200		3950
	Week 18	1400	2900	3600	5200		3250
Vacc 3	Week 24	900	1100	1600	5600		1350
	Week 26	300	1600	1600	4000		1600
	Week 28	400	2700	2900	4500		2800
	Week 30	300	1200	3000	3300		2100
	Week 32	300	1300	2300	5600		1800
	Week 34	300	1200	3900	3000		2100
	Week 36	200	1000	9400	2200		1600
	Week 38	200	900	2300	2300		1600

		G2 CPP					
	Rabbit	M7	M28	F5	F6	F22	median
Vacc 1	Week 0	100	0	0	0	0	0
	Week 2	1300	600	1100	1200	600	1100
	Week 4	5800	Clotted	7600	7100	3300	6450
	Week 6	14000	Clotted	8900	7500	4200	8200
Vacc 2	Week 8	17300	Died	4100	6200	4000	5150
	Week 10	133600		40300	118500	62800	118500
	Week 12	20300		7100	38900	10100	15200
	Week 14	19300		7000	10400	6500	10400
	Week 16	12600		3900	7200	3600	7200
	Week 18	6000		6400	5200	3000	6000
Vacc 3	Week 24	7800		6500	4000	1600	6500
	Week 26	295600		122300	195100	88400	195100
	Week 28	95400		46700	83800	65300	83800
	Week 30	72700		20700	63500	48500	63500
	Week 32	76200		12100	30400	33600	30400
	Week 34	Died		10600	23100	38000	16850
	Week 36			10300	13600	24900	11950
	Week 38			7600	5700	12000	6650

		G4 MMM					
	Rabbit	M8	M24	F14	F20	F31	median
Vacc 1	Week 0	100	0	0	0	0	0
	Week 2	100	0	0	0	100	0
	Week 4	100	0	0	0	0	0
	Week 6	100	300	200	0	100	100
Vacc 2	Week 8	100	500	Died	0	100	100
	Week 10	300	4400		0	6400	300
	Week 12	0	6400		0	1100	550
	Week 14	0	Died		100	2000	50
	Week 16	0			100	1700	50
	Week 18	0			100	1200	50
Vacc 3	Week 24	0			0	1200	0
	Week 26	100			1600	7000	850
	Week 28	0			3300	3200	1650
	Week 30	100			2800	1900	1450
	Week 32	100			2000	1200	1050
	Week 34	100			1500	1200	800
	Week 36	0			400	1000	200
	Week 38	0			1000	900	500

		G5 MPP					
	Rabbit	M9	M15	M18	F29	F30	median
Vacc 1	Week 0	0	0	100	1000	0	0
	Week 2	0	0	0	600	0	0
	Week 4	0	0	100	500	Died	50
	Week 6	400	100	100	1000		250
Vacc 2	Week 8	300	100	100	400		200
	Week 10	31600	1200	10500	49600		21050
	Week 12	6800	14100	7800	4100		7300
	Week 14	6400	4000	9900	2900		5200
	Week 16	5300	2400	10100	2300		3850
	Week 18	6500	1300	5900	2000		3950
Vacc 3	Week 24	2500	1200	4400	1600		2050
	Week 26	94800	210200	79100	21600		86950
	Week 28	75400	103000	34000	21500		54700
	Week 30	20300	40400	47500	13300		30350
	Week 32	22700	28100	21100	11800		21900
	Week 34	19300	30600	18000	5800		18650
	Week 36	8700	20400	9400	6400		9050
	Week 38	2800	36400	11900	2700		7350

		G3 PPP					
	Rabbit	M10	M12	M27	F4	F13	median
Vacc 1	Week 0	0	0	0	100	100	0
	Week 2	400	600	800	2000	3500	800
	Week 4	500	2100	2100	7000	3900	2100
	Week 6	700	2100	2000	4500	4800	2100
Vacc 2	Week 8	1100	3100	1500	4400	2400	2400
	Week 10	41600	98500	43100	96700	63600	65700
	Week 12	40000	22900	34800	27100	39200	34800
	Week 14	15300	9800	32100	10700	17700	13000
	Week 16	10600	8000	35200	39900	12800	22900
	Week 18	15500	4600	12300	26300	9100	13900
Vacc 3	Week 24	6000	1500	5800	8500	4600	5900
	Week 26	356000	52500	69400	55800	150200	62600
	Week 28	64100	33300	40800	Clotted	63300	40800
	Week 30	31300	20200	29400	Clotted	Missing	29400
	Week 32	26600	12200	29300	Died	Died	26600
	Week 34	16400	8600	39300			16400
	Week 36	14600	5600	17300			14600
	Week 38	4100	4700	11400			4700

		G6 CMP					
	Rabbit	M11	M16	F1	F3	F26	median
Vacc 1	Week 0	0	0	68	0	0	0
	Week 2	100	1500	500	400	400	400
	Week 4	500	10800	9100	4100	1600	4100
	Week 6	1100	6700	7900	7000	2700	6700
Vacc 2	Week 8	1600	3500	8200	9300	2500	3500
	Week 10	23700	255200	141900	287500	79400	198550
	Week 12	6700	36200	63900	194100	26500	36200
	Week 14	4100	38300	28800	59900	13700	33550
	Week 16	7000	Died	20300	36400	8600	20300
	Week 18	2500		15200	33400	8400	15200
Vacc 3	Week 24	2500		9700	23100	7600	9700
	Week 26	58900		171000	179400	151600	171000
	Week 28	24300		104800	144400	68500	104800
	Week 30	43800		70700	64100	25600	64100
	Week 32	22600		67900	58900	34100	58900
	Week 34	17200		50400	44400	6900	44400
	Week 36	8800		37500	27400	20500	27400
	Week 38	7600		23900	24400	7800	23900

Columns on the left indicate time of vaccination and sampling, and the column on the right shows the median titre for a particular time point. Titres highlighted in green indicate peak responses after each vaccine administration.
